# Some Proposals for Contributions by Patients to Hospitals

**Published:** 1889-01-26

**Authors:** Nelson Hardy


### Some Proposals for Contributions by Patients to Hospitals.

By Nelson Hardy, F.R.C.S. Edin. (being an abstract of his paper on the subject).

What do we mean by the Pay System in Hospitals? As at present adopted, we mean the payment by out-patients, not of the poorest class, of sums varying from threepence a-week, as at Guy's, or even a penny or twopence elsewhere, to sixpence, one shilling, two shillings, or two shillings and sixpence a week at the various special hospitals for medical advice, supposed to be of a superior quality, and a supply of medicine lasting for a week. It has been fully described, while this paper was in progress, by the secretary to one of the hospitals for diseases of the skin, who had previously been connected with the Hospital Saturday Fund, and who receives, as his predecessors had done, 15 per cent. of the receipts, whether from patients or from the public. The people benefited are said to include out-patients who give nothing to the hospital, out-patients who make monthly payments of three shillings, five shillings, and ten shillings, out-patients who give a shilling or two, and then change into monthly patients. For checking the payments by the out-patients there are books, with counterfoils consecutively numbered. No out-patient paid money without there being a proper counterfoil, so that a proper account could be kept. The receipt was pasted in the patient's book, and the patient, when he went to the doctor, took his book with him.

In the case of in-patients, payments vary from half-a-guinea weekly at one of the hospitals for women for medical and surgical treatment, operations, board and lodging, to five guineas weekly at the establishment for gentlewomen in Harley Street, and seven guineas weekly at the St. Thomas' Home, the patrons of the latter including, it is said, officers of the army and navy, clergymen, etc., all of whom are described as legitimate objects of charity (and no doubt, if we take Sir James Paget's estimate of £2,000 a-year as the income below which it is almost impossible to provide in private families the equivalent of hospital treatment) they may, in a certain sense, be considered to be so). But I suppose that no one will contend that they are exactly the class for which hospitals such as St. Thomas's or Guy's were originally founded or endowed, and that if it were proposed to turn all the wards of an endowed hospital into pay wards—that is, to replace the poor for whom they were intended, and who cannot pay, by those of a superior social position who can—an outcry would probably arise; in fact, the partial adoption of the very same plan is defended on the ground that it enables those hospitals to provide more free beds for the poor. The calculation on which this statement is based seems to me so very curious that I should like to draw attention to it.

In a paper by Mr. Burdett-Coutts, M.P., read before this association in the early part of last year, it is stated about Guy's Hospital at p. 15, on the authority of the medical superintendent of the hospital, that "the cost of an occupied bed amounts to £71 per annum, so that the payments made by patients, £54 10s. (one guinea a week), do not cover their expenses. On the other hand, the arrangement enables the governors to keep a large number of beds open for the reception of free patients." "In this case," adds Mr. Burdett-Coutts, "the twenty beds supply fifteen more beds." Let us work out this little sum, and see if we arrive at the same conclusion:

| | |
|---|---|
| 20 beds costing £71 each ... ... | ... £1,420 |
| Payments by patients, £54 10s. × 20 | ... 1,090 |
| Net loss to hospital | £330 |
| Cost of 15 more beds which this loss of £330 is said to supply—71 × 15 ... ... | ... 1,065 |
| Total loss to hospital on treatment in 20 paying beds and 15 free beds for one year | ... £1,395 |

[Mr. Hardy wholly misses Mr. Burdett-Coutts' point. It is that the present patients treated who now contribute nothing would then increase the hospital's income by their payments £1,090 a-year; that is by £25 more than the cost of occupying 15 of the beds vacant for want of funds. This the table proves to be correct.]

The first proposal for contributions from patients to hospitals which we have to consider to-night, then, is this, that the system of small payments by in- and out-patients, which has been already in practice for some years at the special hospitals, should be adopted at the large general hospitals.

The second proposal to which I shall ask your attention is one by Mr. W. Bousfield, who has devoted so much time and attention to what is known as the Provident System of Medical Relief, in his capacity as chairman of the Metropolitan Provident Medical Association.

With regard to the first proposal, which is no new one, having been advocated for the last twenty years by almost every hospital reformer who has considered the subject, and especially by the large and representative Medical Committee in 1871, which included Sir Spencer Wells and Mr. Timothy Holmes, the late Drs. Anstie, Meadows, and Fairlie Clarke, and many others of equal standing as hospital physicians and surgeons, I am of opinion that, if the medical staffs of our large general hospitals could see their way to get it adopted, it would, even from a financial point of view, be a distinct advantage to the hospitals. If the staffs object that it is necessary to keep the out-patient departments as they are, for the sake of the medical schools, then it becomes a question whether a dozen medical schools are needed in London, and whether it is worth while, for the sake of them, to keep up also a dozen out-patient departments.

The great charm of the second proposal, in my eyes, consists in this: that it openly recognises the truth—which is more or less carefully concealed in other proposed Pay Systems, but which has to be faced nevertheless—that, in order to make any system of cheap physic pay, you must be prepared to compete successfully with the general practitioners, who are already established in superabundant numbers all over London, and amongst whom there is a certain proportion forced by the *res angusta domi* to take a strictly commercial view of the value of medical services—a view which has led to the establishment of those sixpenny dispensaries by which money is to be made, though not, I am afraid, much professional reputation.

This second part of the system has been already partially tried at the Metropolitan Hospital (formerly the Metropolitan Free Hospital), in Kingsland Road, and the experience there does not seem very encouraging for other parts, since one of the first results has been to give rise to a correspondence in the *Lancet* between some practitioners in the neighbourhood of the hospital and the medical officers attached to it, in which the former attack, and the latter defend, this new departure by the hospital authorities.

Ultimately the following protest, which expresses very clearly and forcibly the objections of general practitioners resident in the neighbourhood to the plan, has been drawn up and extensively signed:

"We, the undersigned, medical practitioners resident in the vicinity of the Metropolitan Hospital, Kingsland Road (late Metropolitan Free Hospital), desire to protest against the action taken by the committee of that institution in converting it into a Provident Dispensary, and we do so on the following grounds:

"(1) That the function of a hospital is to relieve the medical wants of the poor, whilst this institution, as at present managed, is of little or no use to the poor in the neighbourhood, its rules being so framed that those who cannot pay after the first attendance are turned away from its doors.

"(2) That this innovation is not a genuine attempt to relieve the poor, but is, as was plainly stated in public meeting by the hon. secretary, an attempt to 'make the thing pay.'

"(3) That the original funds of the hospital were subscribed to benefit the poor, and not the better class of poor, who hitherto have been able to pay their ordinary medical attendant.

"(4) That the plan establishes a low standard of competition, which is detrimental to the practitioners in the neighbourhood (and, by its possible extension, to practitioners in general) as well as to the staff of the hospital. It also has the tendency to lower the appreciation by the public of services rendered to them by the profession.

"(5) A hospital conducted on these commercial principles naturally alienates itself from the support and sympathy of local practitioners."

I will add no word of comment on this protest save this, that as probably each practitioner who has signed it could influence at least, let us say, twenty families to give or withhold subscriptions to hospitals, the loss to the hospital funds through their opposition will probably more than counterbalance the gain from patients' payments.

It would appear, however, that the mere fact of the Metropolitan Hospital having tried this plan, and with alleged success, is likely to induce other and much greater institutions to follow suit; and Sir Edmund Currie, who speaks from a lengthened and intimate acquaintance with the facts of the situation, is sufficiently sanguine to trust to see before long a great revolution effected in the entire hospital system by the general adoption of some scheme whereby steady, thrifty provision during health, as well as during the trouble itself, against sickness, may be encouraged among poor people, to the raising of the standard of their own self-respect, and to the considerable lightening of the burdens now laid upon all charitable medical institutions. But Sir Edmund points out in a letter to the *Times* that, to be at all effective, any such system should be worked from each hospital itself as a centre, and the limits of the district attached to each hospital for medical cases should be clearly defined.

A third and very recent proposal is that of Mr. Algernon Coote, secretary to the Lock Hospital, who suggests that in order to supply the sum of £100,000, at which figure he estimates the annual deficit of the London hospitals, one out of ten of the London population should regularly contribute one penny a week; and he asks, Is it Utopian to suggest that the 500,000 pence per week may be obtained? He refers to the practice in some of the large Scotch towns of contributing regularly one penny per week, and is of opinion that the workshop collections in connexion with the London Hospital Saturday Fund might be largely increased. If the 750,000 working people of London were wisely approached, a very large number of them, he thinks, would contribute. The contributions from the working classes might be supplemented by collections in "Red Cross" one-penny-a-week collecting boxes, for which many would be glad to apply. The 500,000 pence would, he observes, give the hospitals £105,000, and still leave more than 3 per cent. (if required) for the working expenses of the effort.

Ever since, in 1883, at a Social Science Conference held in London, I heard this plan of systematic collections described by those who were actually engaged in them in the provinces, it has struck me that what is wanted in the metropolis is to bring one of these men to London, and set him to work to reproduce here the organisation which has proved so successful in the provinces. But as often as the thought has occurred, the question has also arisen, Under whose auspices could such a work be undertaken with any prospect of success? The organisation of the Hospital Sunday Fund is evidently unsuited to such an attempt. It has, besides, become much too clerical and too completely controlled by the dominating influence of its permanent vice-president, to respond readily to any impulse from without.

The Hospital Saturday Fund, unfortunately, appears to me to be worked largely in the interests of special hospitals, but would otherwise appear to be the most suitable organisation for the purpose—unless, indeed, The Hospitals Association should see its way to take action in the matter, either alone or in conjunction with the Hospital Saturday Fund; but whoever takes the matter up will find, I believe, that a necessary preliminary to any successful working of the scheme would be the organisation of some sort of concerted action between the medical charities, hospitals, and dispensaries in various parts of London.

I do not see how the clinical teaching of our great hospitals is likely to flourish under such a system; nor should I like to defend the present out-patient departments before a committee composed largely of working men, subscribers to hospitals. Any such combination, however, as I have hinted at, of hospitals and dispensaries in each district of London, would itself almost amount to a peaceful revolution; while the mere mention of the medical schools and of the out-patient departments shows what large questions will have to be discussed before this matter can be finally settled, and to settle which it would require, I have long been of opinion, a Royal Commission of Inquiry.

